# A Fault Diagnosis Method of Rotating Machinery Based on One-Dimensional, Self-Normalizing Convolutional Neural Networks

**DOI:** 10.3390/s20143837

**Published:** 2020-07-09

**Authors:** Jingli Yang, Shuangyan Yin, Yongqi Chang, Tianyu Gao

**Affiliations:** School of Electrical Engineering and Automation, Harbin Institute of Technology, Harbin 150080, China; 19S101111@stu.hit.edu.cn (S.Y.); 18S001022@stu.hit.edu.cn (Y.C.); 18B901013@stu.hit.edu.cn (T.G.)

**Keywords:** one-dimensional convolutional neural networks, self-normalizing neural networks, SeLU, α-dropout, fault diagnosis

## Abstract

Aiming at the fault diagnosis issue of rotating machinery, a novel method based on the deep learning theory is presented in this paper. By combining one-dimensional convolutional neural networks (1D-CNN) with self-normalizing neural networks (SNN), the proposed method can achieve high fault identification accuracy in a simple and compact architecture configuration. By taking advantage of the self-normalizing properties of the activation function SeLU, the stability and convergence of the fault diagnosis model are maintained. By introducing α-dropout mechanism twice to regularize the training process, the overfitting problem is resolved and the generalization capability of the model is further improved. The experimental results on the benchmark dataset show that the proposed method possesses high fault identification accuracy and excellent cross-load fault diagnosis capability.

## 1. Introduction

Due to its strong point of versatility, rotating machinery is currently widely adopted in various pieces of mechanical equipment and works in extremely complex environments. Damage will not only impede the normal operation of the equipment, but also cause huge economic losses and pose threats to personal safety. Consequently, performing studies on the fault diagnosis of rotating machinery has become an urgent issue in the field of machine health monitoring.

Generally speaking, the fault diagnosis techniques of rotating machinery are commonly classified into the three types: model-based, signal-based and machine learning-based [1,2]. In model-based methods, a dynamic model should be developed based on the characteristics of vibration responses and the fault generation mechanism of rotating machinery. Cheng et al. [3] established a torsional vibration model for the planetary gear system, and conducted a signal simulation under the normal state and different breaking modes of the sun wheel gear. Then, a new metric named sideband-amplitude ratio was extracted from the frequency spectrum to quantitatively evaluate the damage degree. Park et al. [4] constructed a lumped parametric model of planetary gears based on the relationship between the gear mesh stiffness and the transmission errors, and it was verified in detecting planetary gear failures. Model-based fault diagnosis methods can enhance the comprehension of the transmission behavior and vibration responses of rotating machinery under different failure modes. However, there exist many assumptions and constraints in the model building process; thus, the influences of various uncertainties cannot be fully considered. Furthermore, there is still a certain gap between the simulation and the actual situations, which also has an impact on the fault diagnosis results to some extent. In signal-based methods, two techniques can be used to obtain fault information for the diagnosis of rotating machinery: signal filtering or signal decomposition methods to separate fault components from normal components, and time-frequency analysis methods to reveal the time-varying characteristics of the frequency components of vibration signals. Vibration signals are commonly used in the field of rotating machinery diagnosis because the machine sounds can reflect the most inherent information of health state and contain lots of information, such as working condition [5,6]. Teng et al. [7] extracted the sensitive components of vibration signals using the traditional narrow-band filtering technique at first; then, the inverted spectral analysis and Hilbert-yellow transform were applied to detect the obvious cracking faults; and finally, the Gaussian wavelet transform and multi-scale envelope spectral demodulation were employed to successfully detect the cracking faults in the composite fault mode. Feng et al. [8] combined a Kalman filter with a higher-order energy demodulation operator to effectively diagnose early faults with different signature frequencies. Although fault diagnosis methods based on the signal processing technology are intuitive, practical and physically meaningful, traditional signal decomposition and demodulation methods still have their own limitations. For example, the methods for extracting the envelope spectrum and the transient frequency spectrum components are still complex, as they rely on staff with rich engineering experience to discriminate the types of faults. Recently, the development of machine learning has pushed the fault diagnosis of rotating machinery towards intelligence. In machine learning-based methods, the fault diagnosis process is divided into the two stages [9]: the fault feature extraction stage and the health state recognition stage (i.e., fault classification). The feature extraction aims at extracting information about the health state from the raw signals, removing redundancy and facilitating the fault classification of rotating machinery. The fault classification involves the selection of an appropriate classification method to construct a fault diagnosis model for identifying faults of rotating machinery. Traditional machine learning methods—artificial neural networks (ANN) [10], k-nearest neighbor (KNN) [11], sparse representation-based classifier (SRC) [12,13], support vector machine (SVM) [14,15], etc.—artificially extract fault features from the collected data and select sensitive features for training to achieve fault type identification automatically. Chen et al. [16] utilized probabilistic neural networks to perform effective fault diagnosis of hydroelectric turbine generators. Pandya et al. [17] presented an improved KNN algorithm based on an asymmetric proximity function to enhance the diagnosis precision of bearings. However, these methods still rely on the artificial feature extraction and suffer from problems such as low accuracy and poor generalization performance.

With the emergence of deep learning methods, ground-breaking solutions to the fault diagnosis problem of rotating machinery have been provided recently. Deep learning-based diagnosis approaches apply hierarchical networks to learn abstract fault features layer by layer, and then place an output layer after the last extraction layer to accomplish the fault identification task. Nowadays, depth network models are broadly used in the field of fault diagnosis of rotating machinery, such as depth neural networks (DNN), stacked automatic encoder (SAE), depth belief networks (DBN) and convolutional neural networks (CNN). Jia et al. [18] proposed a five-layer SAE model for the fault diagnosis of rotating machinery; the raw data were converted into the frequency-domain data and subsequently fed it into the model to implement the diagnosis task, and the effectiveness of the method was fully verified by experiments of rotating bearings and planetary gearboxes. Wang et al. [19] introduced the batch normalization layer (BN) into SAE, which could address the issue of internal covariance shifting while training multilayer networks. The experimental results showed that the proposed method could enhance fault identification accuracy and accelerate convergent speed of the training process. Yang et al. [20] presented a DNN-based automatic classification algorithm, which was evaluated by the sub-signals extracted in the frequency-domain. Shen et al. [21] adopted a feature robustness-enhanced adaptive fault diagnosis network based on a deep compression auto-encoder (CAE) and demonstrated its superiority on a gearbox databank. Yin et al. [22] optimized the network structure of DBN by a genetic algorithm and utilized it for the fault diagnosis of gear drive chains. Gan et al. [23] constructed a two-layer fault diagnosis network based on DBN, which adopted a wavelet transform for data preprocessing and implemented fault type and fault degree identification respectively based on the two-layer network. Li et al. [24] combined a hybrid diagnosis model based on SAE and DBN. DBN was used to discriminate health states on the learning features extracted from SAE. Shang et al. [25] published a DBN-based fault diagnosis model for rolling bearings, which could evade the complex structure of deep neural nets to some extent. The proposed model has the merits of easily training and good fault diagnosis capability. However, most fault diagnosis models based on deep learning theory are regular fully-connected networks with low model generalization performance, and the parameters increase exponentially with the number of layers. Therefore, issues of overfitting and decreasing diagnosis ability still affect the actual applications of above methods.

Compared with the fully-connected DNN, CNN’s characteristics such as sparse connection, weight sharing and pooling operations can reduce the number of parameters of the training network, and enhance the model’s stability and generalization performance. The original version of CNN is a two-dimensional structure inspired by the visual system, which has achieved remarkable results in the field of image recognition because of its ability to describe the natural 2D spatial correlations in images [26,27]. Nowadays, the traditional 2D-CNN algorithm has been extensively applied in the field of fault diagnosis. Wen et al. [28] modified the LeNet-5 to perform fault diagnosis for bearings and centrifugal pumps, wherein the raw time-domain signal was transformed into a two-dimensional gray scale image to train the model. Guo et al. [29] adopted a time-frequency domain transformation of the original signal to obtain a continuous wavelet transform scale diagram (CWTS), on whose basis they adopted CNN to directly classify the fault signal. The validity and generality of the proposed method were confirmed on a rotor experiment platform. Zhao et al. [30] developed a planetary gearbox fault diagnosis strategy based on the synchro squeezing transform (SST) and the deep convolutional neural networks (DCNN), which showed the fault identification accuracy was up to 98.3%. Sun et al. [31] converted the raw signal to a 2D image; then they carried out automatic feature extraction from the image via CNN and completed the fault classification of bearings. Inspired by the successful applications in the fields of natural language processing and speech recognition [32,33], the 1D-CNN algorithm was introduced for fault diagnosis only recently. Liu et al. [34] improved the traditional LeNet-5 network, and their results showed that the improved 1D LeNet-5 network could achieve more significant performance on fault diagnosis. Eren et al. [35] utilized a compact adaptive 1D-CNN classifier to implement the fault diagnosis of induction bearings and proved the feasibility of the algorithm on a real dataset. Zhang et al. [36] presented an end-to-end fault diagnosis approach based on 1D-CNN, and the experimental results exhibited high accuracy, even in a noisy environment. Several studies have been conducted to prove the 1D-CNN has advantages over 2D-CNN in processing vibration signals for fault diagnosis. An et al. [37] compared the performances of 2D-CNN and 1D-CNN in bearing fault diagnosis, which demonstrated that the 1D-CNN model offered better feature extraction capability than the 2D-CNN model. Jing et al. [38] compared the fault diagnosis performances of different CNN models under three types of input data (i.e., the raw data, the spectrum data and the combined time-frequency data), and the results indicated that the 1D-CNN model is superior than others when the spectrum data is adopted as its input.

To be noted is that most of the current studies focusing on fault diagnosis methods of rotating machinery have been conducted on ideal data—that is, assuming the training samples cover the total working conditions of rotating machinery. However, in real engineering situations, it is infeasible to obtain the ideal data for all working conditions of rotating machinery because the working load changes constantly. Therefore, it is critical to utilize the data collected from limited conditions to establish fault diagnosis models. In other words, how to enhance the generalization capability of fault diagnosis models has emerged as one of the hot spots in practical industry applications.

**Figure 1:**
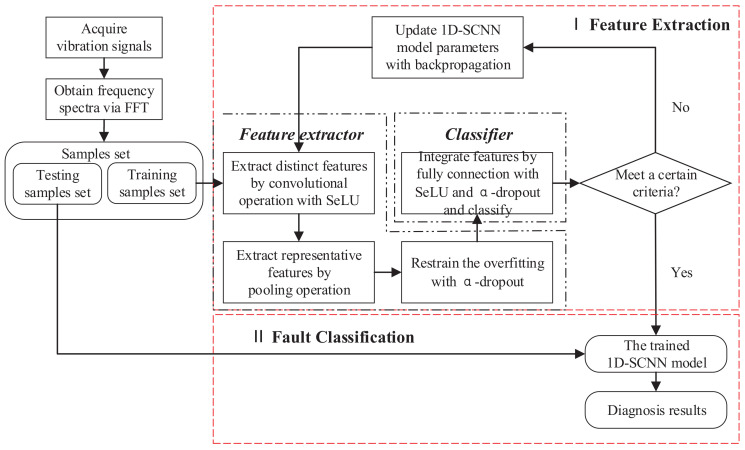
The block diagram of the proposed method.

Aiming at the shortfalls of existing methods, this paper proposes a fault diagnosis method based on one-dimensional, self-normalizing convolutional neural networks (1D-SCNN) to address the low accuracy and poor generalization capability of fault diagnosis for rotating machinery. As shown in Figure 1, the main innovation of this paper is summarized as follows.

1. A fault diagnosis model based on 1D-SCNN is presented, which has a simple and compact architecture configuration with only a convolutional layer and a pooling layer. Compared with the conventional techniques, it can achieve competitive performance in terms of fault diagnosis accuracy and generalization capability.
2. The scaled exponential linear units (SeLU) are employed to strengthen the features of the fault signal. With the self-normalizing properties, activations can maintain normalization when propagating through layers of the network. Therefore, SeLU can maintain the stability and convergence of the network, and enhance the generalization capability of the model.
3. The α-dropout algorithm is introduced into the feature extractor and classifier simultaneously, which not only can restrain the overfitting at the initial stage of training, but also should be able to accelerate the speed of the network’s parameters updating and further boost the generalization capability of the model.
4. A series of experiments utilizing the Case Western Reserve University bearing dataset are conducted. The results demonstrate that the proposed method possesses good fault diagnosis accuracy and generalization capability, and provides an excellent solution for enhancing the reliability and maintainability of mechanical equipment.

## 2. One-Dimensional Convolutional Neural Networks

The one-dimensional convolutional neural network (1D-CNN) is a type of depth-feedback neural network with convolutional kernels of unit width. The literature [39] claims that CNNs dedicated to classification tasks each consist primarily of a feature extractor and a classifier.

### 2.1. Feature Extractor

The feature extractor is a composite structure used to map the raw signal into the feature space to characterize diverse fault features, and it is an essential element of 1D-CNN. The feature extractor is composed of the one-dimensional convolutional layer, the activation layer and the pooling layer.

(1) One-dimensional convolutional layer

The one-dimensional convolutional layer extracts distinct fault features from the input data (or output features of the previous layer) through convolutional operations. It involves multiple feature maps with many neurons, and each neuron is sparsely connected to the feature map of the upper layer by a weight matrix (convolutional kernels). CNN weights are shared when the input feature map is the same as the output. Sparse connection and weight sharing can reduce network complexity and avoid the overfitting problem. The convolutional operation is shown in Equation (1) [36].

(1) $$y^{l(i,j)}=K_{i}^{l}*x^{l\left(r^{j}\right)}=\sum_{j^{\prime }=0}^{W-1}K_{i}^{l\left(j^{\prime }\right)}x^{l\left(j+j^{\prime }\right)}$$

where $K_{i}^{l\left(j^{\prime }\right)}$ denotes the $j^{\prime }$-th weight or weights (if the depth of kernel is larger than 1) of the *i*-th convolutional kernel in the *l*-th convolutional layer, $x^{l\left(r^{j}\right)}$ denotes the local region where the *j*-th convolutional kernel in the *l*-th convolutional layer is convolved and *W* is the width of the convolutional kernel.

(2) Activation layer

After the convolutional calculation, the activation function is adopted to perform a nonlinear transition, which can make it easier to distinguish the features of different types of faults. Rectified linear unit function (ReLU) is the commonly adopted activation function. The expression is given by the Equation (2) [36].

(2) $$a^{l(i,j)}=f\left(y^{l(i,j)}\right)=\max\left\{0,y^{l(i,j)}\right\}$$

where $a^{l(i,j)}$ is the activation of $y^{l(i,j)}$.

(3) Pooling layer

The pooling layer conducts feature dimension reduction to achieve more representative characteristics. The pooling operation can enhance the stability and robustness of the features by eliminating the noise interference [26]. The mean-pooling layer and max-pooling layer are extensively utilized in convolutional networks. Max-pooling performs the local max operation on the perception domain of the output feature map, which is calculated by Equation (3) [36].

(3) $$p^{l(i,j)}=\max_{(j-1)W+1\leq t\leq jW}\left\{a^{l(i,t)}\right\}$$

where $a^{l(i,t)}$ denotes the activation of the *t*-th neuron in the *i*-th frame of the *l*-th layer, *W* is the width of the pooling region and *j* denotes the *j*-th pooling kernel.

### 2.2. Classifier

The classifier involves full-connection layers and activation functions that integrate and classify fault features derived from the output of the pooling layer. Notably, the activation function for the hidden layer is ReLU, and for the last layer is softmax, as shown by Equation (4) [36]. Through softmax regression, distinct fault features are converted into a normalized probability distribution, and the closer the value to 1, the more probable it is a true fault type.

(4) $$P\left(j\right)=\frac{e^{Z^{l}\left(j\right)}}{\sum_{k=1}^{n}e^{Z^{l}\left(k\right)}}$$

where $Z^{l}\left(j\right)$ denotes the input of the *j*-th node in the *l*-th full-connection layer.

## 3. Methodology

### 3.1. Overview

**Figure 2:**
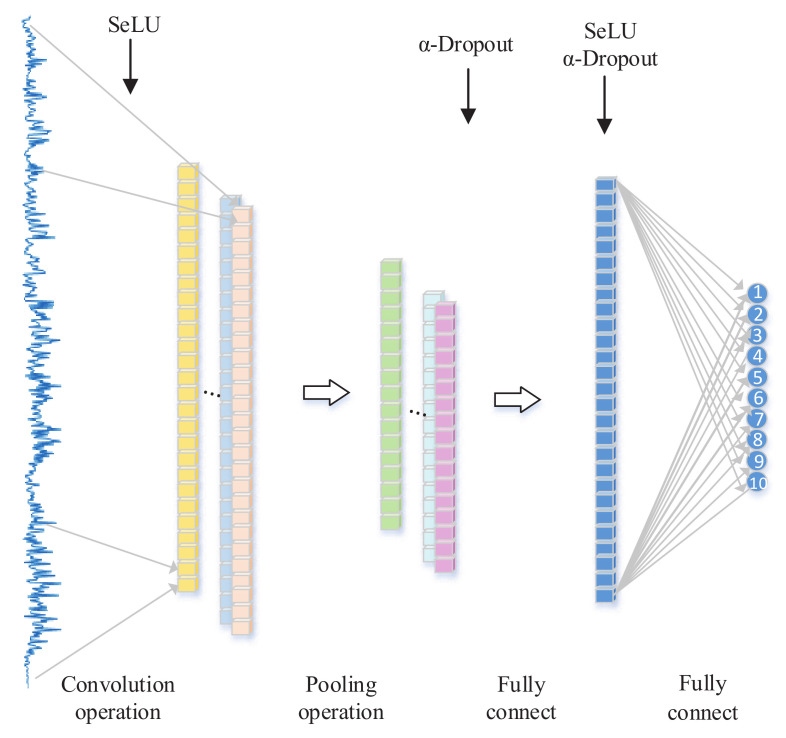
The architecture of the proposed 1D-SCNN method for fault diagnosis.

The proposed 1D-SCNN-based fault diagnosis method is concerned with increasing the fault diagnosis accuracy of rotating machinery and further promoting the generalization capability of fault diagnosis models, which can achieve fault type identification of rotating machinery even under non-ideal conditions. The architecture of the proposed method for fault diagnosis is shown in Figure 2.

The activation function SeLU is introduced in the convolutional layer. With the input feature of zero mean and unit variance, SeLU ensures that the output feature still has zero mean and unit variance. Therefore, it can make the network robust and avoid the overfitting problem. Details about the convolutional layer-based strategy are illustrated in Section 3.2.

Adding an α-dropout layer after the pooling layer can improve the generalization capability of the model, wherein α-dropout is improved by normalizing the output features upon dropout. Details about the pooling layer-based strategy are shown in Section 3.3.

Applying the activation functions SeLU and α-dropout to the full-connection layer can not only enhance the fault features, but also further improve the generalization capability of the model. Details about the full-connection layer-based strategy are described in Section 3.4.

### 3.2. SeLU-Based Enhancement of Convolutional Fault Feature Extraction

**Figure 3:**
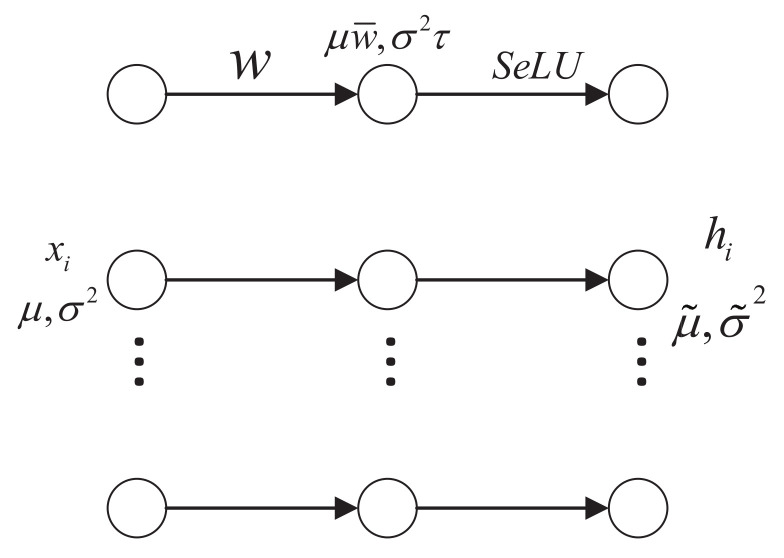
The block diagram of SNN.

The structure of the self-normalizing neural networks (SNN) is shown in Figure 3. Assuming ${\left\{x_{i}\right\}}_{i=1}^{n}$ is the training set of SNN, the sample $x_{i}$ of the random variable *X* obeys a distribution with the mean μ and variance $\sigma^{2}$. The linear transformation of $x_{i}$ yields the pre-activation vector $z_{i}=w_{i}x_{i}$, where $w_{i}$ is the weight matrix and $z_{i}$ is the implementation of the random variable *Z*. The mean and variance of *Z* are shown by Equations (5) and (6).

(5) $$E\left(Z\right)=\sum_{i=1}^{n}w_{i}E\left(x_{i}\right)=\sum_{i=1}^{n}w_{i}\mu =\mu \bar{w}$$

(6) $$\mathrm{Var}\left(Z\right)=\mathrm{Var}\left(\sum_{i=1}^{n}w_{i}x_{i}\right)=\sum_{i=1}^{n}w_{i}^{2}\sigma^{2}=\sigma^{2}\tau$$

where $\bar{\omega }=\sum_{i=1}^{n}w_{i}$ and $\tau =\sum_{i=1}^{n}w_{i}^{2}$. According to the central limit theorem, *Z* obeys the normal distribution: $N(\mu \bar{w},\sqrt{\sigma^{2}\tau })$ with $p_{N}\left(Z;\mu \bar{w},\sqrt{\sigma^{2}\tau }\right)$.

Klambauer [40] et al. presented a novel activation function named SeLU as follows.

(7) $$selu\left(x\right)=\lambda \left\{\begin{array}{ll} x, & \mathrm{if}x>0\\ \alpha e^{x}-\alpha , & \mathrm{if}x\leq 0 \end{array}\right.$$

The function performs the mapping of the mean and variance of the random variable from the former layer to the next layer by mapping *F* [40]:

(8) $$F:\left(\begin{array}{c} \mu \\ \sigma^{2} \end{array}\right)\mapsto \left(\begin{array}{c} \tilde{\mu }\\ {\tilde{\sigma }}^{2} \end{array}\right):\begin{array}{c} \tilde{\mu }\left(\mu ,\bar{w},\sigma^{2},\tau \right)=\int_{-\infty }^{\infty }selu\left(z\right)p_{N}\left(z;\mu \bar{w},\sqrt{\sigma^{2}\tau }\right)dz\\ {\tilde{\sigma }}^{2}\left(\mu ,\bar{w},\sigma^{2},\tau \right)=\int_{-\infty }^{\infty }selu{\left(z\right)}^{2}p_{N}\left(z;\mu \bar{w},\sqrt{\sigma^{2}\tau }\right)dz-{\left(\tilde{\mu }\right)}^{2} \end{array}$$

After normalizing the weight matrix: $\bar{w}=0,\tau =1$, given the fixed point $\left(\mu ,\sigma^{2}\right)=\left(0,1\right)$, we have $\tilde{\mu }=\mu =0,{\tilde{\sigma }}^{2}=\sigma^{2}=1$. By solving Equation (8) for α and λ, the solutions α=1.6732632423543772… and λ=1.0507009873554804… can be obtained. At this point, the (2 × 2)-Jacobia of *F* [40] is

(9) $$J\left(\mu ,\sigma^{2}\right)=\left(\begin{array}{cc} \partial \frac{\mu^{new}\left(\mu ,\sigma^{2}\right)}{\partial \mu } & \partial \frac{\mu^{new}\left(\mu ,\sigma^{2}\right)}{\partial \sigma^{2}}\\ \partial \frac{\nu^{new}\left(\mu ,\sigma^{2}\right)}{\partial \mu } & \partial \frac{\nu^{new}\left(\mu ,\sigma^{2}\right)}{\partial \sigma^{2}} \end{array}\right),J\left(0,1\right)=\left(\begin{array}{cc} 0.0 & 0.088834\\ 0.0 & 0.782648 \end{array}\right)$$

The norm of *J* is equal to 0.7877 < 1; i.e., the mapping *F* is a contraction mapping and the corresponding fixed point is stable.

As mentioned above, SeLU can ensure that the activation passes through the network layers in a normalized state, and its output value tends to a stable fixed point. Once the perturbation and noise occur, the shift will be pulled back to the normalized state immediately, which can avoid the overfitting problem and offer good robustness. Compared with batch normalization, there is no need to calculate the variance and offset of the current input data before updating the network parameters, which means the reduction of the computational complexity. Furthermore, SeLU has no dead zone; i.e., neurons can still be activated when the input is less than 0, which means richer features can be obtained compared to ReLU. Consequently, applying the activation function SeLU to the convolutional layer of the 1D-SCNN model can incorporate the advantages of self-normalization properties into the feature extractor of 1D-SCNN, and enhance the feature extraction capability of the model. Moreover, the robustness of the network can also be guaranteed.

### 3.3. α-Dropout-Based Improvement of Pooling Layer Generalization Capability

Dropout is an effective technique to address the overfitting problem of deep neural nets [41], the key idea of which is to randomly drop units and their connections from the neural network to prevent co-adapting between units. The dropout rate q(0<q<1) indicates the probability that each neuron in the network layer will stop working, and its value is generally set to be 0.5.

To enhance the generalization capability of the model, 1D-SCNN introduces the idea of dropout by adding it after the pooling layer. Furthermore, the α-dropout improved algorithm is adopted to maintain the normalization of the values that pass across the network. In the dropout algorithm, the activations are scaled by 1/q during the training process and remain constant during the testing process. Moreover, the dropout variable *d* obeys a binomial distribution B(1,q). α-Dropout can set the input to $\alpha^{\prime }$ because the default value of the low variance is $\lim_{x\to -\infty }\mathrm{sel}u\left(x\right)=-\lambda \alpha =\alpha^{\prime }$, where the mean and variance are described as follows [40].

(10) $$\begin{array}{l} E\left(xd+\alpha^{\prime }\left(1-d\right)\right)=q\mu +\left(1-q\right)\alpha^{\prime }\\ \mathrm{Var}\left(xd+\alpha^{\prime }\left(1-d\right)\right)=q\left(\left(1-q\right){\left(\alpha^{\prime }-\mu \right)}^{2}+\nu \right) \end{array}$$

To ensure that the variance and mean remain constant after applying the α-dropout algorithm, the affine transformation shown in Equation (11) [40] is adopted.

(11) $$\begin{array}{l} E\left(a\left(xd+\alpha^{\prime }\left(1-d\right)\right)+b\right)=\mu \\ \mathrm{Var}\left(a\left(xd+\alpha^{\prime }\left(1-d\right)\right)+b\right)=\nu \end{array}$$

When μ=0,ν=1, $a={\left(q+\alpha^{\prime 2}q\left(1-q\right)\right)}^{-1/2},\;b=-{\left(q+\alpha^{\prime 2}q\left(1-q\right)\right)}^{-1/2}\left(\left(1-q\right)\alpha^{\prime }\right)$, the values of the parameters *a* and *b* depend on *q* and $\alpha^{\prime }$.

In the absence of the α-dropout algorithm, the forward propagation process for each hidden layer parameters is described by Equation (12).

(12) $$\begin{array}{l} z_{i}^{\left(l+1\right)}=w_{i}^{\left(l+1\right)}y^{l}+b_{i}^{\left(l+1\right)}\\ y_{i}^{\left(l+1\right)}=selu\left(z_{i}^{\left(l+1\right)}\right) \end{array}$$

With the α-dropout algorithm, the forward propagation process of each hidden layer parameter is shown in Equation (13).

(13) $$\begin{array}{l} r_{j}^{\left(l\right)}∼\mathrm{Bernoulli}\left(q\right)\\ {\tilde{y}}^{\left(l\right)}=r^{\left(l\right)}\times y^{\left(l\right)}\\ z_{i}^{\left(l+1\right)}=w_{i}^{\left(l+1\right)}{\tilde{y}}^{\left(l\right)}+b_{i}^{\left(l+1\right)}\\ y_{i}^{\left(l+1\right)}=selu\left(z_{i}^{\left(l+1\right)}\right) \end{array}$$

where $z_{i}^{\left(l+1\right)}$ denotes the input of the (l+1)-th layer and $y_{i}^{\left(l+1\right)}$ denotes the output of the (l+1)-th layer. $r^{\left(l\right)}$ is a vector of the *l*-th layer, which consists of binomially distributed random variables with values of 1−q.

In short, the overfitting problem of 1D-SCNN can be addressed by applying the α-dropout algorithm to the pooling layer of the feature extractor, and the generalization capability of the model can also be improved.

### 3.4. SeLU and α-Dropout-Based Advancement of Full-Connection Layer Generalization Capability

The full-connection layer is used to identify the fault features output by the feature extractor. The selection of the activation function plays a crucial role in the linear separability of the features and the stability of the network. Therefore, SeLU is selected as the activation function of the full-connection hidden layer, the principles and characteristics of which are described in Section 3.2. Moreover, to further improve the generalization capability of the model, the full-connection layer is improved via the α-dropout algorithm, the principles and characteristics of which are detailed in Section 3.3.

### 3.5. Training of 1D-SCNN

The 1D-SCNN training process includes the forward propagation of the signal and the backward propagation of the error. The probability distribution of the sample type is obtained by the forward propagation of the signal, and the cross-entropy loss function is employed to evaluate the consistency of this output with the real sample label. Assuming that $q_{k}^{j}$ represents the probability of the *k*-th sample predicted to be *j*-th class and $p_{k}^{j}$ is the one-hot vector of the target sample label, the expression of the cross-entropy loss function is given by Equation (14) [36].

(14) $$L=-\frac{1}{m}\sum_{k=1}^{m}\sum_{j}p_{k}^{j}\log q_{k}^{j}$$

where *m* denotes the size of the mini-batch. The backward propagation of the error is the main foundation for 1D-SCNN to optimize the weights, which takes the loss value as the source of the error and updates the gradient from back to front by the chain law.

The error backpropagation begins at the full-connection layer and is calculated similarly to ANN, and the process can be demonstrated by Equations (15)–(19) [36]. Here, $z^{i+1(j)}$ is the logits value of the last layer.

(15) $$\frac{\partial L}{\partial z^{i+1(j)}}=\sum_{k=1}^{m}p_{k}^{j}q_{k}^{j}-p_{k}^{j}$$

(16) $$\frac{\partial L}{\partial W_{ij}^{l}}=\frac{\partial L}{\partial z^{i+1(j)}}\cdot \frac{\partial z^{i+1(j)}}{\partial W_{ij}^{l}}=\frac{\partial L}{\partial z^{i+1(j)}}\cdot a^{i(i)}$$

(17) $$\frac{\partial L}{\partial b_{j}^{l}}=\frac{\partial L}{\partial z^{l+1(j)}}\cdot \frac{\partial z^{l+1(j)}}{\partial b_{j}^{l}}=\frac{\partial L}{\partial z^{l+1(j)}}$$

(18) $$\frac{\partial L}{\partial a^{l(i)}}=\sum_{j}\frac{\partial L}{\partial z^{l+1(j)}}\cdot \frac{\partial z^{l+1(j)}}{\partial a^{l(i)}}=\sum_{j}\frac{\partial L}{\partial z^{l+1(j)}}\cdot W_{ij}^{l}$$

(19) $$L=-\frac{1}{m}\sum_{k=1}^{m}\sum_{j}p_{k}^{j}\log q_{k}^{j}$$

The weight W and bias b are updated iteratively as follows.

(20) $$\begin{array}{l} W_{ij}^{l}=W_{ij}^{l}-a\frac{\partial L}{\partial W_{ij}^{l}}\\ b_{i}^{l}=b_{i}^{l}-a\frac{\partial L}{\partial b_{i}^{l}} \end{array}$$

where *a* denotes the iteration rate.

The backpropagation calculation of the pooling layer is the inverse operation of the forward propagation. As for the max-pooling operation, only pooling units that deliver the maximum are involved. The specific propagation process is shown in the Equation (21) [36].

(21) $$\frac{\partial L}{\partial a^{l(i,t)}}=\frac{\partial L}{\partial p^{l(i,j)}}\cdot \frac{\partial p^{l(i,j)}}{\partial a^{l(i,z)}}=\left\{\begin{array}{cc} 0 & t\neq t_{h}\\ \frac{\partial L}{\partial p^{l(i,j)}} & t=t_{h} \end{array}\right.$$

where *h* indicates the maximal location of the pooling region.

The backpropagation calculation of the convolutional layer requires the gradient of each unit, which is calculated as follows.

(22) $$\frac{\partial L}{\partial y^{l(i,j)}}=\frac{\partial L}{\partial a^{l(i,j)}}\cdot \frac{\partial a^{l(i,j)}}{\partial y^{l(i,t)}}=\left\{\begin{array}{cc} 0 & y^{l(i,j)}\leq 0\\ \frac{\partial L}{\partial a^{l(i,j)}} & y^{l(i,j)}>0 \end{array}\right.$$

(23) $$\frac{\partial L}{\partial x^{l(j)}}=\sum_{i}\frac{\partial L}{\partial y^{l(i,j)}}\cdot \frac{\partial y^{l(i,j)}}{\partial x^{l(j)}}=\sum_{i}\frac{\partial L}{\partial y^{l(i,j)}}\cdot \sum_{j^{\prime }=0}^{W-1}K_{i}^{l}\left(j^{\prime }\right)$$

(24) $$\frac{\partial L}{\partial K_{i}^{l}\left(j^{\prime }\right)}=\frac{\partial L}{\partial y^{l(i,j)}}\cdot \frac{\partial y^{l(i,j)}}{\partial K_{i}^{l}\left(j^{\prime }\right)}=\frac{\partial L}{\partial y^{l(i,j)}}\cdot \sum_{j}x^{l(j)}$$

### 3.6. Fault Diagnosis Process

**Figure 4:**
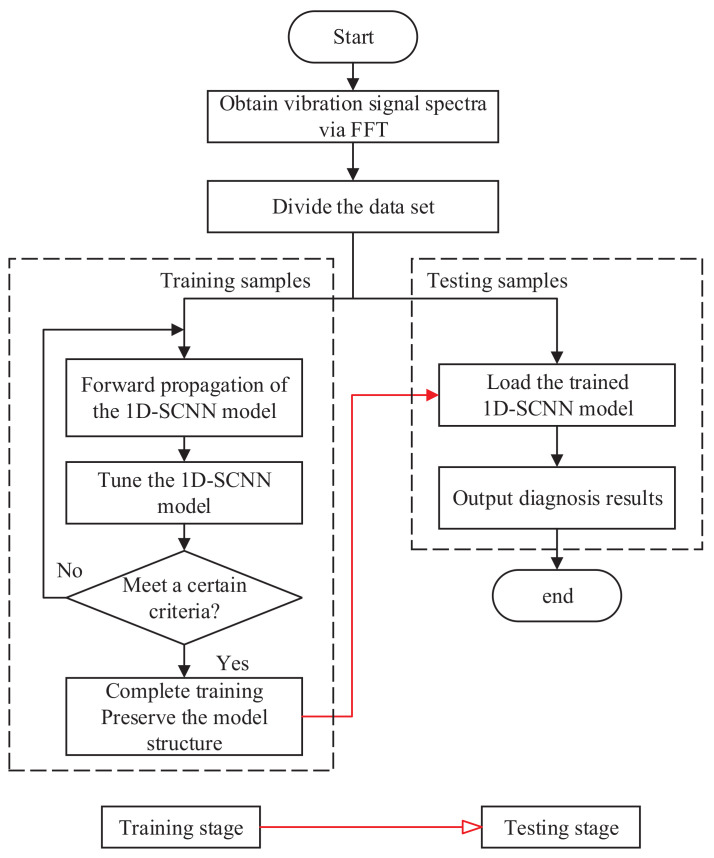
Flow diagram of 1D-SCNN-based fault diagnosis method of rotating machinery.

The flow of the proposed 1D-SCNN-based fault diagnosis method of rotating machinery is shown in Figure 4. The method can adaptively extract fault features from the spectrum of the vibration signals, thereby enabling effective fault identification by features. Besides, the proposed fault diagnosis model has superior generalization capability which in turn provides good adaptive performance under cross-load levels. The algorithm is implemented as follows.

1. The spectra of the vibration signals are obtained using the fast Fourier transform (FFT) at the raw signal length without the windowing function, and are used as input samples for the 1D-SCNN model.
2. Randomly divide the input samples into a training sample set and a testing sample set with a ratio of 7:3. The training sample set serve as the input for the training stage of the model and the testing sample set is adopted for the testing stage.
3. Train the 1D-SCNN model by the forward propagation and the backward propagation operations, and save the trained model after meeting a certain criterion.
4. Load the trained fault diagnosis model and input the testing sample set into above diagnosis model to obtain the fault diagnosis results.

## 4. Experimental Results and Analysis

The deep learning framework chosen in this study was Keras. By using Python as the programming language, and adopting Pycharm as the editor, the proposed method was developed with TensorFlow. The computer used during the experiments was configured with CPU i5-8265U, 16 G memory and a 64-bit operating system. To verify the superior performance of the proposed 1D-SCNN-based fault diagnosis method, experiments for evaluating of the fault diagnosis accuracy and the generalization capability were conducted, respectively.

### 4.1. Dataset Description

Rolling bearings from the Bearing Laboratory at Case Western Reserve University (USA) were selected as experimental subjects. In this paper, the vibration signal data of a deep groove ball bearing at the driven end with a sampling frequency of 12 kHz are selected for experimental analysis. The damaged parts of the bearing are: inner race, ball and outer race; the degrees of failure are 0.007, 0.014, 0.021 (unit: inch), respectively. The healthy state is labeled as 1, and labels for the inner race’s damaged states are 2, 3 and 4, in ascending order of degree of damage; the ball and outer race states are labeled in the same way. In other words, above 10 labels represent the 10 health states of the bearing, i.e., fault types. The four datasets A, B, C and D were prepared with load levels of 0, 1, 2 and 3 (unit: hp), respectively. Each dataset contains 10 fault types described above, and each fault type consists of 100 samples. Furthermore, each sample involves 1024 consecutive data points, which were obtained by dividing the spectrum. Specific information on the experimental dataset is illustrated in Table 1.

### 4.2. Results Analysis

#### 4.2.1. Selection of Model Parameters

The hyperparameters of the 1D-SCNN model are set as follows: the convolutional kernel size is initialized by the LeCun uniform initializer (ensure that the output mean is 0 and the variance is 1), the pooling kernel size is 2, the pooling step length is 2, the number of neurons in the full-connection layer is 64, the α-dropout rate is 0.5, the optimizer is Adam, the iteration number is set to 200 and the sample size for each training round is 100. The size and step length of convolutional kernels, and the numbers of convolutional kernels and layers play vital roles in the performance of the model, which was determined experimentally in this study.

The preliminary evaluation experiments on the fault diagnosis accuracy and generalization capability demonstrate that above three hyperparameters have few effects on the fault diagnosis accuracy under the same load level, whereas the model generalization capability under the cross-load level conditions is more affected. Accordingly, the average of the fault identification accuracy under cross-load level conditions is employed as the indicator for parameter optimization. To eliminate the influence of random factors, 30 groups of experiments are repeated.

(1) Convolutional kernel size and step length

**Figure 5:**
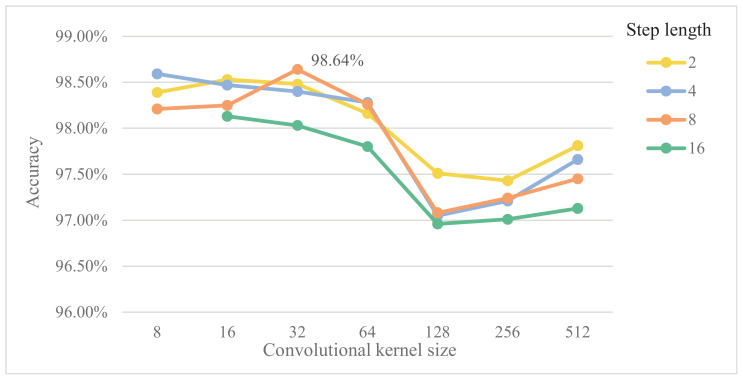
Effects of the convolutional kernel size and step length on the performance of 1D-SCNN.

Zhang et al. [36] stated that the first layer of a convolutional network should have large convolutional kernels for one-dimensional signals, which facilitates the automatic learning of network-oriented fault diagnosis features. Considering that the length of the data sample selected in this study was 1024, the effects of convolutional kernel size and step length on the generalization capability of the model are discussed with convolutional kernel sizes of 8, 16, 32, 64, 128, 256 and 512, respectively. First, the numbers of convolutional and pooling layers were fixed both to one with 16 convolutional kernels, and then the step length is multiplied from 2 to 16 under each convolutional kernel, where the maximum step length is 8 when the convolutional kernel is 8. Thus, the effects of convolutional kernel size and step length on the diagnosis accuracy are analyzed simultaneously. The experimental results are depicted in Figure 5.

As revealed by Figure 5, the impact of convolutional kernel size on diagnosis performance is greater than that of convolutional step length, which reflects the significance of convolutional kernel size selection in the first layer of the model. Since the features extracted from small convolutional kernels are not representative enough, large convolutional kernels can cause the computational volume to spike. In this paper, the convolutional kernel size of the first layer of is set to 32 and the step length is set to 8.

(2) Number of convolutional kernels

After determining the convolutional kernel size and the step length, the effects of the number of convolutional kernels on the generalization capability of the 1D-SCNN model are analyzed, and the experimental results are shown in Table 2.

As shown in Table 2, as the number of convolutional kernels increases from 8 to 32, and the accuracy rises initially and falls later, and achieves the highest value when the number of convolutional kernels is 16; thus, the number of convolutional kernels of the first layer is selected as 16.

(3) Number of layers

The number of convolutional kernels, the convolutional step length and the number of convolutional kernels were determined, after which the effects of alternating layers of convolutional and pooling on the generalization capability of the 1D-SCNN model were analyzed. Drawing on the design principle of the network structure in the literature [42], the first layer was devised as a large convolutional kernel, and all other layers adopted a convolutional kernel size of 3 × 1. The results of the experiment are displayed in Table 3.

As shown in Table 3, the fault diagnosis accuracy shows a decreasing trend as the number of network layers increases; i.e., the model possesses the best generalization capability when it contains only a single layer. Therefore, we utilizes one layer for the proposed model.

Based on above experimental results, the parameters of the 1D-SCNN model are determined as shown in Table 4. The model has only one convolutional and pooling layer with the convolutional kernel size of 32 and the step length of 8. The number of units in the full-connection layer is 64, and the softmax layer yields 10 outputs, which corresponds to 10 bearing health states.

#### 4.2.2. Fault Diagnosis Accuracy Assessment

This section evaluates the fault diagnosis accuracy of the proposed model by describing a series of experiments using four datasets, A, B, C and D, respectively. For each dataset, 30 random sample experiments were conducted under same load level. During each experiment, 70 samples of each fault type were randomly selected as training samples, and the remaining were used as testing samples. In other words, the size of the training sample set was 700 and that of the testing sample set was 300. Furthermore, the average of the fault identification accuracy was taken to eliminate the influence of random factors.

**Figure 6:**
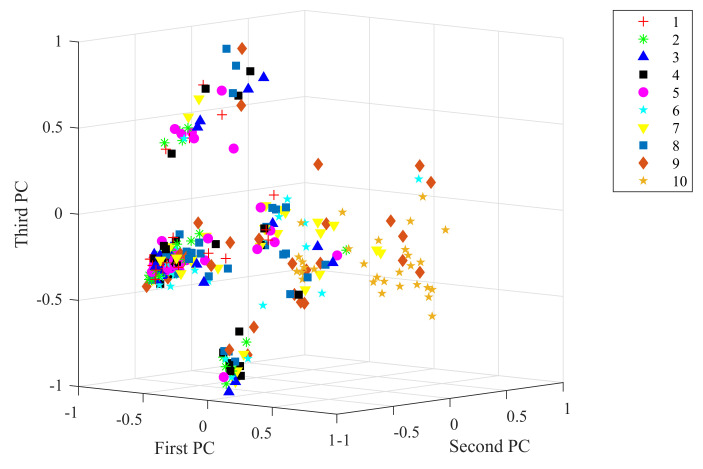
Raw vibration signals.

**Figure 7:**
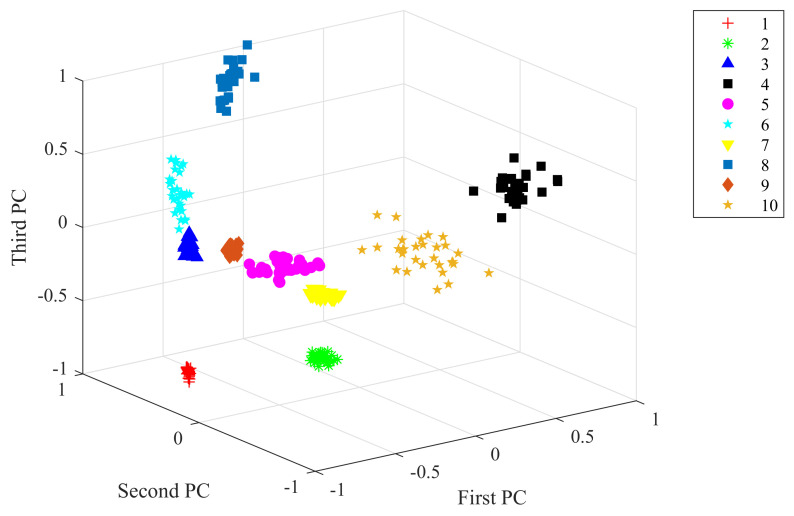
Features extracted from 1D-SCNN.

Taking the data under 2 hp load level as an example, the raw vibration signal and the feature extracted from the 1D-SCNN model were processed by principal component analysis (PCA). With visualizing techniques, the obtained top three principal components for the raw vibration signal and the extracted feature achieved by the feature extractor of the 1D-SCNN model are plotted in Figure 6 and Figure 7, respectively. As depicted in Figure 6, the principal components of the raw vibration signal of the 10 different types of faults exhibit scattered overlap state. In contrast, Figure 7 depicts that the principal components of the extracted feature achieved by the 1D-SCNN model distinctly present non-overlapping discrete states in 3D space. Accordingly, the 1D-SCNN model possesses excellent adaptive fault feature extraction capability.

**Figure 8:**
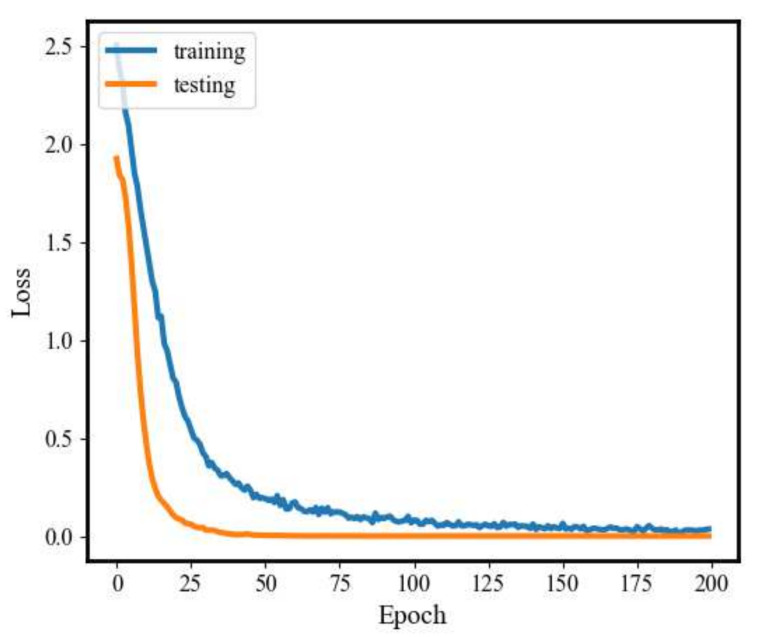
1D-SCNN model loss.

**Figure 9:**
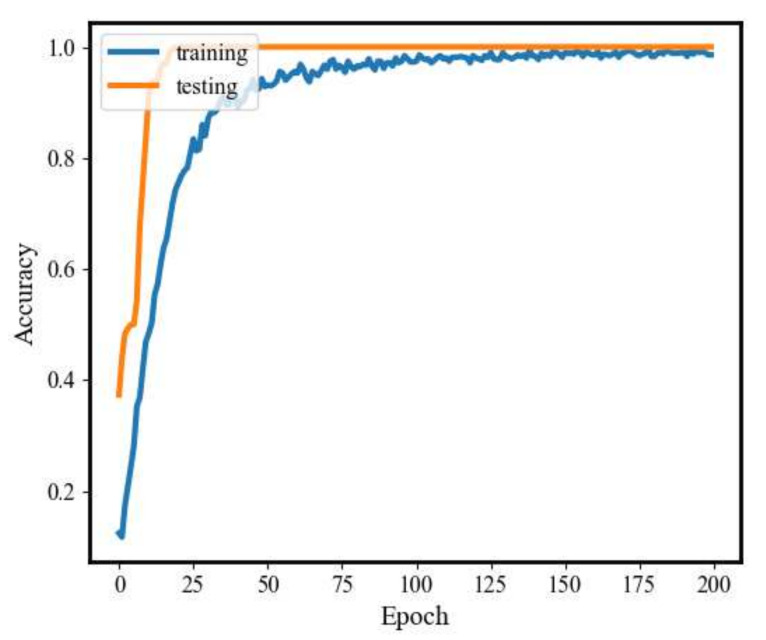
Fault diagnosis accuracy of 1D-SCNN.

Figure 8 and Figure 9 illustrate the changes in model loss and accuracy during the training process and the testing process under 0 hp load level. As shown in these figures, the loss of both the training process and testing process gradually decreases and converges to 0 when the number of iterations reaches about 200. In addition, the training accuracy increases along with the testing accuracy approaching 100% and stabilizes after several iterations.

To further evaluate the fault diagnosis performance of the proposed model, some representative methods in the field of fault diagnosis of rotating machinery (i.e., EMD+SVM [14], FFT+DNN [20], FFT+SDAE [18] and 1D-CNN [34]) were also implemented in these experiments. Among them, the number of neurons per layer was 1024, 800, 200, 200 or 10 for DNN; the number 1024, 800, 200 or 10 for SDAE. In addition, the numbers of mini-batches and iterations were set to 100 and 200, respectively. As shown in Table 5, the diagnosis performance of the traditional machine learning method (EMD+SVM) was worse than those of depth neural networks. Moreover, the 1D-CNN method does not perform well because its input is the time-domain signal, and its optimization time is usually longer than methods that adopt the frequency spectrum as input. Therefore, the 1D-CNN fault diagnosis model is unstable under the specified number of iterations. Including the proposed method, the fault identification accuracy of the three deep learning-based fault diagnosis model is close to 100%, which exhibits superior fault diagnosis performance.

**Figure 10:**
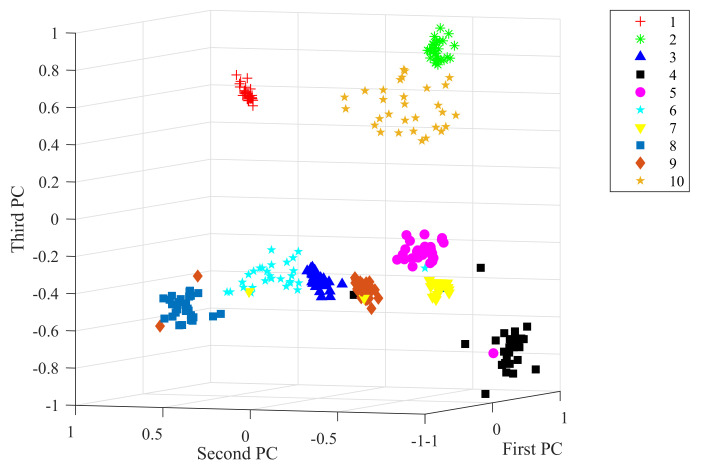
Features extracted by the model under cross-load level conditions.

In the actual applications of rotating machinery, the working conditions are complex and variable, which makes it impossible to obtain adequate data samples under each load level; thus, the diagnosis capability under cross-load level conditions is also an extremely significant performance indicator. The generalization capability of the proposed 1D-SCNN model is evaluated in this section. For each load level of the rolling bearing, 30 fault diagnosis models were generated by the random sample method. During the generation of each model, 70 samples of each fault type under the same load level were randomly selected as training samples. In other words, the size of the training sample set for each diagnosis model was 700. To validate the generalization capability of the generated fault diagnosis models, a series of experiments were conducted for each fault diagnosis model on three testing datasets under different load levels. Over the course of each experiment, 30 samples of each fault type under each load level were selected to form a testing sample set. In other words, the size of the testing sample set was 900, and the mean of the fault identification accuracy was taken to eliminate the influences of random factors. Let us take the 1D-SCNN model under 0 hp load level as an example. The model is used to identify data samples at 2 hp load level. By utilizing PCA on the features extracted from the 1D-SCNN model, the top three principal components obtained are visualized and shown in Figure 10. As shown in the figure, via the feature extraction operation of the 1D-SCNN model, the principal components of the 10 different types of faults under the cross-load level conditions exhibit a non-overlapping discrete state in 3D space, which means fault identification can be accomplished accurately with these extracted features.

#### 4.2.3. Model Generalization Capability Assessment

**Figure 11:**
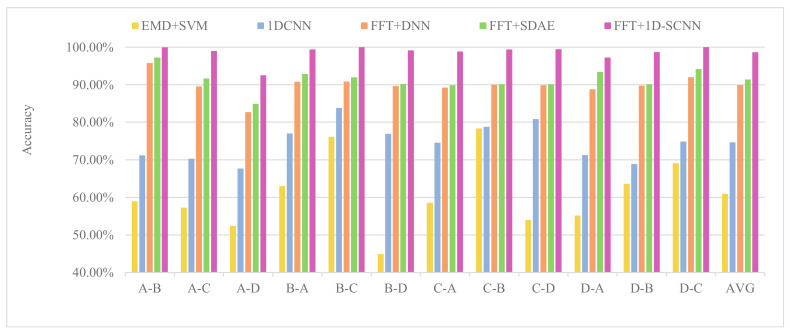
Fault diagnosis accuracy of different methods under cross-load level conditions.

To quantitatively assess the generalization capability of the proposed method, the fault identification accuracy of the proposed 1D-SCNN-based fault diagnosis method is compared with that of some representative methods, such as EMD+SVM, FFT+DNN, FFT+SDAE and 1DCNN under cross-load level conditions. The experimental results are exhibited in Figure 11.

As shown in Figure 11, the proposed 1D-SCNN model achieves the fault identification accuracy of more than 90% under each cross-load diagnosis scenarios, and it offers the best performance among all methods. Furthermore, the training model generated under a certain load level always shows high performance in the identification of samples collected under similar load levels, and its fault identification accuracy also decreases gradually as the load level span increases. The reason for above phenomenon is that the fault samples collected under near-load level have more similarities; i.e., 0 hp causes a similar degree of damage to 1 hp and gaps to 3 hp. Overall, the 1D-SCNN-based fault diagnosis method has an average fault identification accuracy of 98.64%, which is considerably higher than its counterparts. Therefore, it has excellent generalization capability and is suitable for cross-load fault diagnosis.

#### 4.2.4. Influence of SeLU and α-Dropout on Model Performance

**Figure 12:**
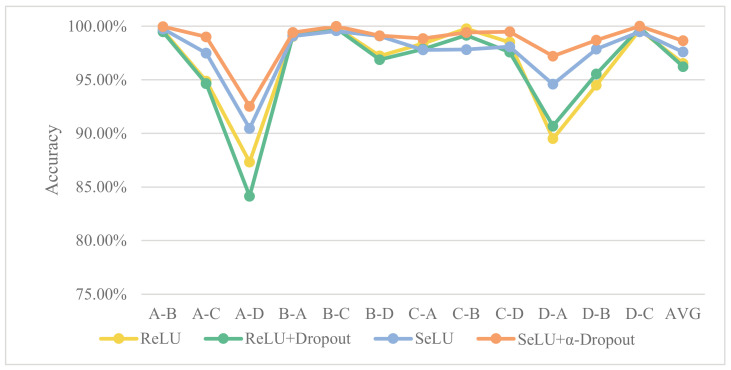
Effects of SeLU and α-dropout on the generalization capability of 1D-SCNN.

This section verifies the enhancement level of SeLU and α-dropout on the performance of the 1D-SCNN model. Based on 1D-SCNN, the performance of the model is verified for four cases; i.e., the activation function is ReLU without dropout, the activation function is ReLU with dropout, the activation function is SeLU without α-dropout and the activation function is SeLU with α-dropout. The experimental results are presented in Table 6 and Figure 12.

It can be seen that both changing the activation function from ReLU to SeLU and using a combination of SeLU and α-dropout have few effects on model diagnosis performance under the same load level.

Figure 12 discusses the effects of SeLU and α-dropout on the generalization capability of the 1D-SCNN model. Overall, the enhancing strategy of changing the activation function from ReLU to SeLU can improve fault identification accuracy of the model under cross-load level conditions to some extent. Combining ReLU with dropout offers no performance improvement over ReLU separately. By contrast, the combination of SeLU and α-dropout provides a significant boost to the performance of fault diagnosis model. Consequently, it is confirmed that SeLU and α-dropout can significantly enhance the generalization capability of the 1D-SCNN model.

## 5. Conclusions

To improve the fault identification accuracy of rotating machinery under cross-load level conditions, a 1D-SCNN-based fault diagnosis method is proposed. The method involves feature extraction based on the frequency spectrum of the raw vibration signals. By introducing the self-normalizing properties of SNN into 1D-CNN through SeLU, and applying α-dropout twice to regularize the model, the accuracy and generalization capability of the fault diagnosis model are greatly enhanced. Furthermore, the model is configured with a simple and compact architecture, which has a good computational complexity. The experimental results demonstrate that the proposed method not only can possesses high fault identification accuracy under the same load level, but also should be able to achieve good performance under cross-load level conditions. Given that in actual engineering situations, it is tricky to obtain ideal data under all working conditions of rotating machinery due to constant changes in speed and load level, the proposed method can utilize the data collected from limited working conditions to build a fault diagnosis model to identify fault types under other working conditions, which has significant engineering application value.

It should be noted that the actual running conditions of the rotating machinery are very complex; thus, it is impossible to obtain a training dataset including all the conditions. Considering the diversity of real engineering situations, future work will be devoted to taking more validation scenarios into account to test the generalization capability of the proposed method. For example, training the model using the datasets of A and C, and then testing the performance of the model using the datasets of B and D. Furthermore, most time-domain features and the simple frequency spectrum are insensitive for bearing fault diagnosis in actual situations. Considering the complexity of real engineering situations, we will try some other features, such as the squared envelope or the squared envelope spectrum, in our future work.

## Figures and Tables

**Table 1:** Description of the experimental dataset.

Damaged Parts		None	Inner Race			Ball			Outer Race			Load
Label		1	2	3	4	5	6	7	8	9	10
Damaged degree		0	0.007	0.014	0.021	0.007	0.014	0.021	0.007	0.014	0.021
A	train	70	70	70	70	70	70	70	70	70	70	0
	test	30	30	30	30	30	30	30	30	30	30	
B	train	70	70	70	70	70	70	70	70	70	70	1
	test	30	30	30	30	30	30	30	30	30	30	
C	train	70	70	70	70	70	70	70	70	70	70	2
	test	30	30	30	30	30	30	30	30	30	30	
D	train	70	70	70	70	70	70	70	70	70	70	3
	test	30	30	30	30	30	30	30	30	30	30	

**Table 2:** Effect of the number of convolutional kernels on the performance of 1D-SCNN.

Number of Convolutional Kernels	8	16	32	64
Accuracy	98.51%	98.64%	98.54%	98.44%

**Table 3:** Effects of layer number on the performance of 1D-SCNN.

Network Structure	Monolayer	Bilayer	Trilayer	Four Layers	Five Layers
Accuracy	98.64%	97.6%	96.37%	93.62%	91.84%

**Table 4:** Parameters of the 1D-SCNN model.

Number	Network Layer	Kernel Size/Step Length	Number of Kernels	Output Size
1	Convolution	32 × 1/8 × 1	16	128 × 16
2	Pooling	2 × 1/2 × 1	16	64 × 16
3	Full-connection	64	1	64 × 1
4	Softmax	10	1	10

**Table 5:** Comparison of experimental results of different methods.

Comparison Method	A	B	C	D	AVG
EMD+SVM	80.90%	84.77%	93.27%	96.57%	88.88%
1D-CNN	92.59%	85.22%	91.29%	92.82%	90.48%
FFT+DNN	100%	100%	100%	100%	100%
FFT+SDAE	100%	100%	100%	100%	100%
FFT+1D-SCNN	99.81%	100%	100%	100%	99.95%

**Table 6:** Effects of SeLU and α-dropout on the diagnostic performance of 1D-SCNN.

Comparison Method	A	B	C	D	AVG
ReLU	99.78%	100%	100%	100%	99.95%
ReLU + Dropout	99.82%	100%	100%	100%	99.96%
SeLU	99.90%	100%	100%	100%	99.95%
SeLU + α-Dropout	99.81%	100%	100%	100%	99.95%
